# COVID-19 seeding time and doubling time model: an early epidemic risk assessment tool

**DOI:** 10.1186/s40249-020-00685-4

**Published:** 2020-06-23

**Authors:** Lei Zhou, Jiang-Mei Liu, Xiao-Ping Dong, Jennifer M. McGoogan, Zun-You Wu

**Affiliations:** 1 Public Health Emergency Center, Chinese Center for Disease Control and Prevention, 155 Changbai Road, Changping District, Beijing, 102206 China; 2 National Center for Chronic and Noncommunicable Disease Control and Prevention, Chinese Center for Disease Control and Prevention, 155 Changbai Road, Changping District, Beijing, 102206 China; 3 Global Health Center, Chinese Center for Disease Control and Prevention, 155 Changbai Road, Changping District, Beijing, 102206 China; 4 National Center for AIDS/STD Control and Prevention, Chinese Center for Disease Control and Prevention, 155 Changbai Road, Changping District, Beijing, 102206 China

**Keywords:** Seeding time, Doubling time, Case report, Risk assessment, SARS-CoV-2, COVID-19

## Abstract

**Background:**

As COVID-19 makes its way around the globe, each nation must decide when and how to respond. Yet many knowledge gaps persist, and many countries lack the capacity to develop complex models to assess risk and response. This paper aimed to meet this need by developing a model that uses case reporting data as input and provides a four-tiered risk assessment output.

**Methods:**

We used publicly available, country/territory level case reporting data to determine median seeding number, mean seeding time (ST), and several measures of mean doubling time (DT) for COVID-19. We then structured our model as a coordinate plane with ST on the x-axis, DT on the y-axis, and mean ST and mean DT dividing the plane into four quadrants, each assigned a risk level. Sensitivity analysis was performed and countries/territories early in their outbreaks were assessed for risk.

**Results:**

Our main finding was that among 45 countries/territories evaluated, 87% were at high risk for their outbreaks entering a rapid growth phase epidemic. We furthermore found that the model was sensitive to changes in DT, and that these changes were consistent with what is officially known of cases reported and control strategies implemented in those countries.

**Conclusions:**

Our main finding is that the ST/DT Model can be used to produce meaningful assessments of the risk of escalation in country/territory-level COVID-19 epidemics using only case reporting data. Our model can help support timely, decisive action at the national level as leaders and other decision makers face of the serious public health threat that is COVID-19.

## Background

Novel coronavirus disease 2019 (COVID-19) causes a serious public health problem worldwide in early 2020 [1–4]. The World Health Organization (WHO) declared the COVID-19 epidemic a “public health emergency of international concern” on 30 January 2020 [5], and then elevated COVID-19 to pandemic status on 11 March 2020 [6]. By 8 May 2020, more than 3.7 million cases and nearly 260 000 deaths had been reported to WHO [7].

The need for situational awareness and predictions of the course of the epidemic have driven an enormous volume of research in just a few short months. Many have used mathematical modelling methods to estimate key epidemiological features, such as basic reproductive number (R_0_), to help inform decision makers so that they can design, evaluate, and adjust control strategies and containment measures [8–11]. However, persistent gaps in our knowledge of COVID-19 has made mathematical modelling challenging [12]. Although many epidemiological parameters affect the potential risk of infectious disease spread in a country (e.g., characteristics of cases, probability of exposure and infection, demographic features of the population, control measures implemented), the essential element is always the exact number of cases. The impacts of all other relevant factors are embodied in the change of the number of cases over time (assuming there is adequate capability for case detection, reporting, and confirmation), making case reporting data a critical input to public health response development [13]. Furthermore, case reporting data is also the timeliest and most easily obtainable data for all countries. So, in theory, effective use of such data could provide a method for evaluating risk for the purpose of informing country-level strategic and tactical decision making.

Therefore, to minimize the impact of knowledge gaps while still meeting the need for timely, convenient, and accurate, yet easy to understand, risk assessments for COVID-19 epidemics at the national level, we aimed to develop a simple, intuitive coordinate model using case reporting data. We furthermore aimed to conduct sensitivity analyses to verify and validate the model, ensuring the risk assessments it produced were meaningful. We call our model the seeding time and doubling time model (i.e., the ST/DT Model).

## Methods

### Data source and analysis

All data used to determine model parameters and to conduct risk assessments using our ST/DT Model were extracted from daily situation reports published by the World Health Organization (WHO; https://www.who.int/emergencies/diseases/novel-coronavirus-2019/situation-reports/). All case reporting data at the individual country level (i.e., dates and cumulative numbers of cases; see Additional file 1: Table S1 ) were analyzed and presented graphically using Excel software (Microsoft 365 version, Microsoft Corp., Redmond, Washington, USA).

### Country selection

Countries to be used in the determination of seeding number (SN) were selected on the basis of their meeting either of two inclusion criteria: (a) having a cumulative 5000 or more cases reported as of 31 March 2020 or (b) having at least 40 days of case reporting data and at least 100 cases accumulated between the date of the first case report in the country and 31 March 2020. A smaller random sample of these selected countries was used in the calculation of mean seeding time (ST) and mean doubling time (DT). A separate sample of 45 countries and territories to be used to conduct early epidemic risk assessments using the ST/DT Model were randomly selected based on one inclusion criterion: having a cumulative number of case reports between 100 and 500, indicating that the country/territory was in the early stage of its epidemic. China was excluded from all analyses since few cases were being reported during this study timespan [7]. Finally, the same 20 countries used to generate mean ST and mean DT were also used in the sensitivity analysis conducted to verify that the model could detect changes in conditions and alter risk assessment outputs. Moreover, 1 of the 20 countries used for mean ST and DT and 1 of the 45 countries used to generate risk assessment were selected (based upon their having detailed published information on their epidemic response measures) to be used in the sensitivity analysis conducted to validate that the changes in risk assessments the model can detect are meaningful based upon what is known of their epidemics and response measures.

All country and territory naming and categorization by region was aligned with WHO situation reports (https://www.who.int/emergencies/diseases/novel-coronavirus-2019/situation-reports/).

### Model structure

In our seeding time and doubling time (ST/DT) Model, only two major epidemiologic parameters were set specifically for each country—seeding time (ST) and doubling time (DT). ST is the time interval, measured in days, between the date of the first case report in a country (i.e., the country’s index case) and the date on which the cumulative number of confirmed cases reached the seeding number (SN). SN is the total number of cases required to “hatch” an epidemic in a country. It can determine the original introduced risk at the beginning of an outbreak in a country and it influences DT. DT is the time interval, measured in days, required to double the total cumulative number of cases, and it can be an indicator of the effectiveness of control measures. Together, ST and DT can be used to determine the risk of an outbreak “taking off,” meaning entering a phase where the numbers of cases grow very rapidly.

**Fig. 1:**
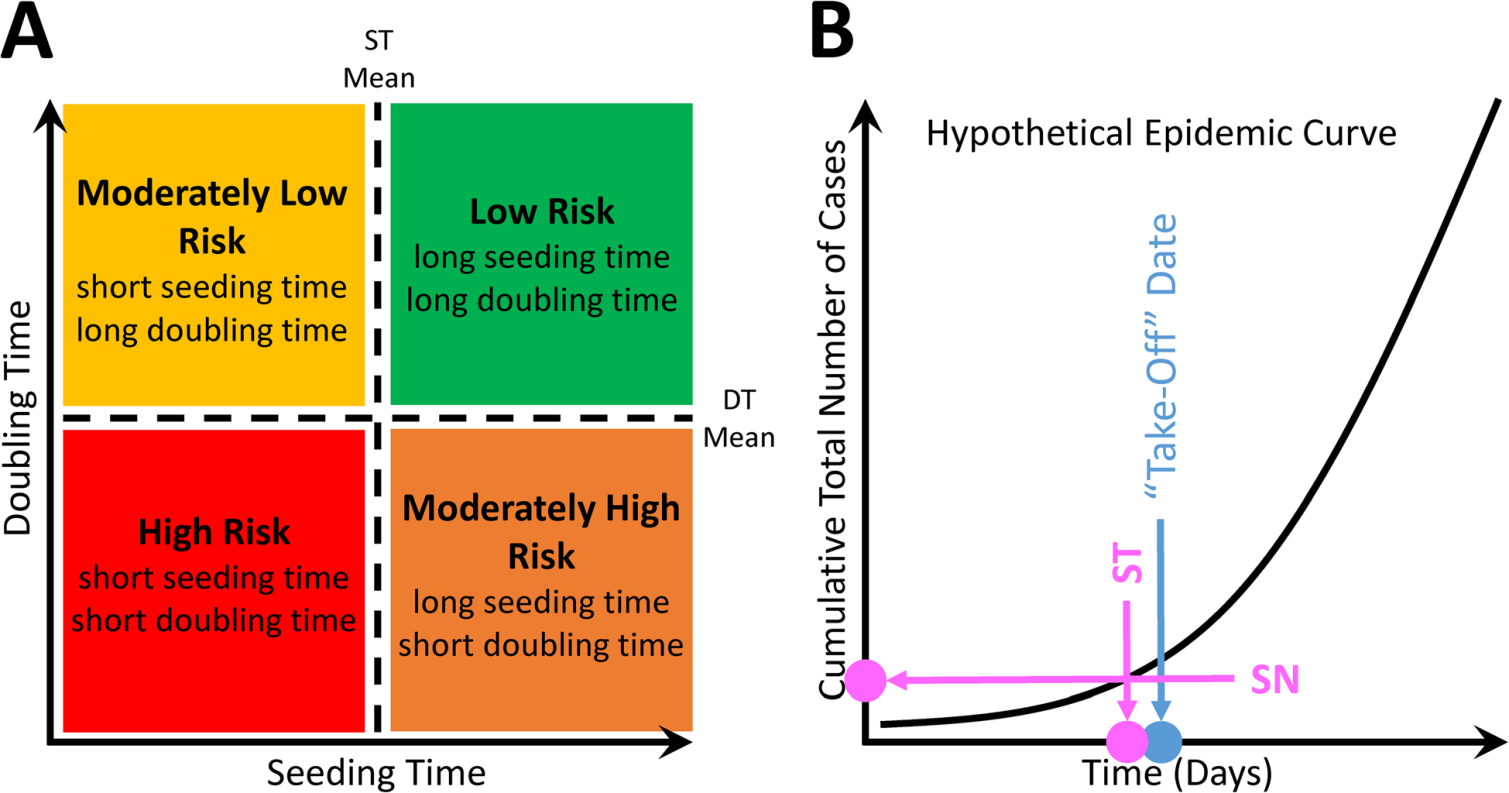
Structure of the ST/DT Model for epidemic risk assessment and method for calculating seeding number (SN) and seeding time (ST). **a** The structure of the ST/DT Model is illustrated by a coordinate plane with ST plotted in days on the x-axis and doubling time (DT) plotted in days on the y-axis. Dashed lines indicate mean ST and mean DT. The four quadrants created are ascribed risk levels—short ST and DT indicate high risk (red), long ST and short DT indicate moderately high risk (orange), short ST and long DT indicate moderately low risk (yellow), and long ST and DT indicate low risk (green). **b** On a hypothetical epidemic curve the date at which the epidemic appears to “take-off” was determined independently by two authors (blue). The number of cumulative cases on the day before is the SN and the date on which the SN is reached is the ST (pink)

The ST/DT Model is illustrated in Fig. 1a. ST increases from short to long on the x-axis while DT increases from short to long on the y-axis. Plotting lines that represent mean ST and mean DT creates four quadrants upon which countries’ epidemics can be plotted. The four quadrants indicate different levels of risk—short ST and DT indicate high risk (red) compared to the low risk indicated by long ST and long DT (green). In between these high and low risk states, are long ST and short DT, ascribed moderately high risk, and short ST and long DT, ascribed moderately low risk. Since DT is more important than ST to the future of epidemics already seeded, the long ST, short DT condition is given a higher risk label than short ST and long DT.

### Determining seeding number

Since both ST and DT depend on SN, we first determined SN. To do this, the epidemiologic curves (plotted as time in days on the x-axis versus cumulative total number of cases on the y-axis) of several countries were assessed by two authors. The two authors independently selected the date on which each epidemic curve appeared to “take-off” (Fig. 1b). Curves for which the two authors did not agree were assessed by a third author and discussed with the research team until consensus was reached on a date. The cumulative number of cases reported up to the day before this “take-off” date was then determined (Fig. 1b). The SN for each of these countries was then used to determine a median SN to be used in the structuring of the ST/DT Model.

### Setting mean seeding and doubling times

To set mean ST and mean DT in order to parse the coordinate plane for the ST/DT Model into the four quadrants, a subset of the countries used in the determination of median SN were used. Each country’s ST was calculated as the time in days it took to reach the median SN. The ST for each of these countries was then used to determine an overall mean ST to be used in the structuring of the ST/DT Model. Early epidemic stage DT for each of the countries in this same subset was calculated as the mean number of days required to double the number of cases from the SN to 2 × SN, then to 4 × SN, and then to 8 × SN cases. The mean DT for each of these countries’ first three doubling periods was then used to determine an overall mean DT to be used in the structuring of the ST/DT Model.

### Sensitivity analyses

Since the model is meant to be used repeatedly by countries to determine how their risk assessment changes over time based on evolving conditions such as implementation of control measures, we sought to assess the model’s sensitivity to these changes using the same subset of countries used for determining mean ST and DT. While ST remains unchanged, shortening or lengthening of DT should reflect changed conditions, thereby allowing countries, ideally, to move from high risk to moderately low risk or from moderately high risk to low risk.

#### Verification

To verify that the ST/DT Model can indeed detect changes and alter resulting risk assessment, later epidemic stage DT was calculated as the mean number of days for each country to observe case doubling (after reaching 8 × SN cases) to 16 × SN, then to 32 × SN, and finally to 64 × SN cases. Later epidemic stage positioning of each country on the ST/DT Model coordinate plane was compared to earlier stage positioning.

#### Validation

To validate that the changes in risk assessment that the ST/DT Model detected were actually meaningful and made sense in light of what was known about changing conditions in the countries, the relative change in position of countries (early to later stage) was compared to what was known about those countries’ outbreaks and response efforts. One country (Australia) was selected from this group of 20 for further analysis. Also, one country (Belarus) out of the 45 used to conduct risk assessments (see below) was also used for further analysis. In both cases, changes in risk assessments over time were compared to timing of changes in epidemic factors and response measures.

### Assessing risk using the ST/DT Model

To use the ST/DT Model to assess epidemic risk at the national level, countries must determine their ST and DT. Using SN, ST is the time, in days, it takes to accumulate enough cases to reach the SN and the initial DT is the time required to double the SN. Initially, the mean of the first few DTs for the country should be used. Each country’s ST and DT is then plotted on the coordinate plane (Fig. 1a) to yield a risk assessment. We used the ST/DT Model to assess risk in countries that were still early in their outbreaks. SN, ST, and early epidemic DT (i.e., mean of the first two or three DTs) were assessed independently by two authors for a sample of countries and territories. Disagreements were then evaluated by a third author and discussed until consensus was reached. All countries were then categorized by region and plotted on the ST/DT Model coordinate plane.

## Results

### Seeding number

**Fig. 2:**
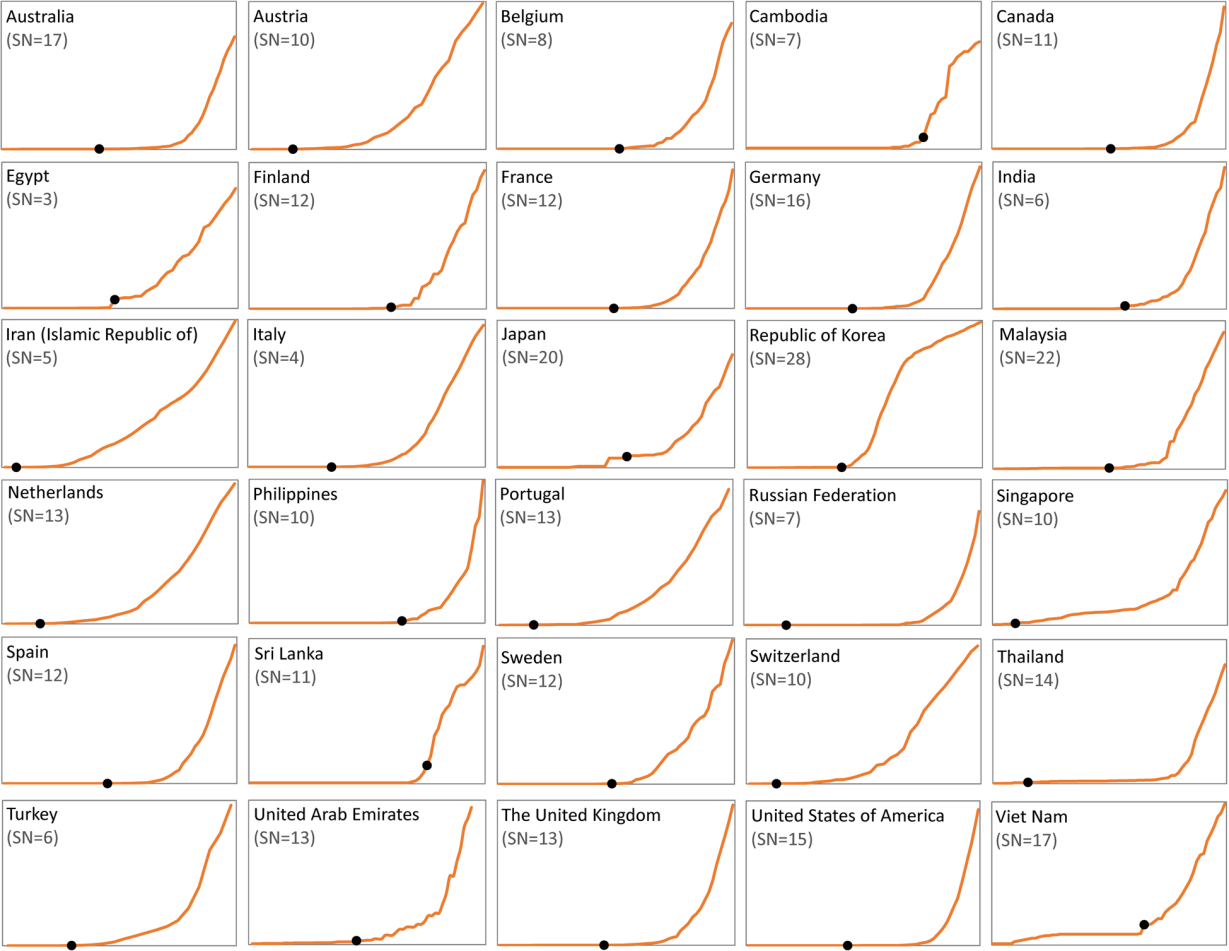
Epidemiologic curves of 30 countries used to determine seeding number (SN). All case report data through 31 March 2020 were used. Multiple researchers independently determined the epidemic “take-off” point (black circle) and date. The total cumulative cases on the day prior to the “take-off” date determined that country’s SN. The median SN for these 30 countries was used in the ST/DT Model

A total of 30 countries met the criteria for inclusion in the determination of median SN. These 30 countries’ epidemiologic curves, epidemic “take-off” points, and individual SNs are shown in Fig. 2. The overall median SN for the 30 countries was 12 days (range: 3–28; interquartile range [IQR]: 10–17). Hence, the SN used for the ST/DT Model was set to 12 cases. Raw and summarized data used to calculate overall median SN can be found in in the Additional file 1: Table S2 and Table S3 .

### Mean seeding time and mean doubling time

**Fig. 3:**
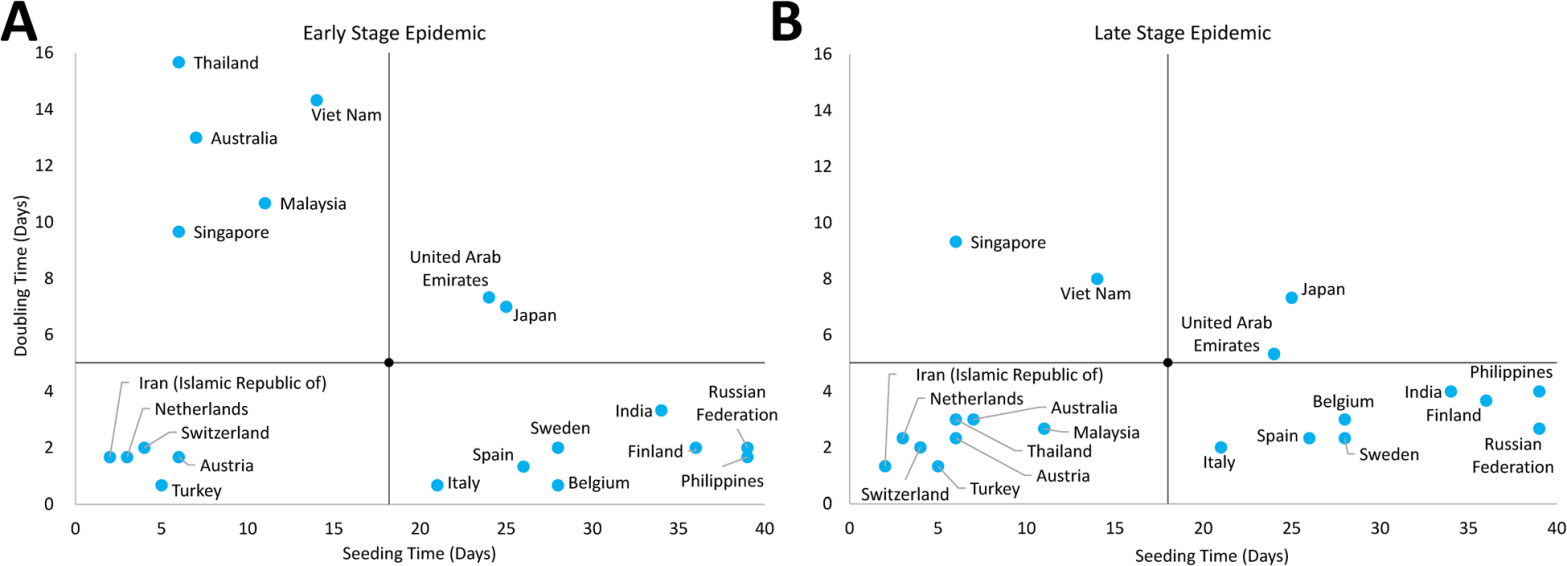
Determination of mean seeding time (ST) and mean doubling time (DT) and ST/DT Model sensitivity analysis. **a** With seeding number (SN) set to 12 cases for all countries and seeding time (ST) for each country calculated as the number of days required to reach SN = 12, early epidemic stage doubling time (DT) for each country was calculated as the mean number of days required to observe case doubling to 24, 48, and then 96 cases. All 20 countries were plotted on the ST/DT Model coordinate plane and overall mean ST was found to be 18 days (vertical line) and overall mean DT was found to be 5 days (horizontal line). **b** For sensitivity analysis, later epidemic stage DT was calculated as the mean number of days for each country to observe case doubling to 192, 384, and 768 cases. The countries with the largest changes from early to later stage epidemic were Australia, Malaysia, and Thailand, all of which moved from moderately low risk to high risk. Viet Nam also had a marked reduction in DT but remained moderately low risk. All countries in the moderately high risk quadrant moved closer to the mean DT line but did not cross over into the low risk quadrant. The only country that did not move at all (ie, had no change in DT) was Switzerland

Among these 30 countries, a total of 20 were used to determine mean ST and mean DT. Each individual country’s ST was first calculated, using SN set to 12 cases, as the time in days it took for the country to accumulate 12 cases beginning from the date of the first case. Each country’s DT was then calculated as the mean of its first three DTs (i.e., time from 12 cases to 24, from 24 to 48, and from 48 to 96). Each country’s ST and mean DT were then plotted on the ST/DT Model’s coordinate plane (Fig. 3a). The overall mean of the 20 countries’ individual ST values was calculated to be 18 days (range: 2–39). So, for the ST/DT Model, mean ST was set to 18 days as shown by the black vertical line on the ST/DT Model (Fig. 3a). The overall mean of the 20 countries’ individual mean DT values was calculated to be 5 days (range: 0.67–15.67). So, for the ST/DT Model, mean DT was set to 5 days as shown by the black horizontal line on the ST/DT Model (Fig. 3a). Raw and summarized data used to calculate country-specific ST and DT and overall mean ST and mean DT can be found in the Additional file 1: Table S2 and Table S3.

### Model verification (results of sensitivity analyses)

Results of the verification portion of the sensitivity analyses are shown in Fig. 3. When the same 20 countries were plotted on the ST/DT Model coordinate plane early in their epidemics (Fig. 3a) and later in their epidemics (Fig. 3b), changes in position are observable for most countries. For example, notably large changes occurred for Thailand (DT shortened by 12.7 days), Australia (DT shortened by 10.0 days), Malaysia (DT shortened by 8.0 days), and Viet Nam (DT shortened by 6.3 days) such that all except Viet Nam moved from the moderately low risk quadrant to the high risk quadrant. Although none of the countries in the moderately high risk quadrant moved into the low risk quadrant, several moved closer—Italy’s DT lengthened by 1.3 days, Belgium’s DT lengthened by 2.3 days, and the Philippines DT lengthened by 2.3 days. The only country that did not move was Switzerland, which had a DT of 2.0 days both early and later in its epidemic. Raw and summarized data used to conduct the sensitivity analysis can be found in the Additional file 1: Table S2 and Table S3 .

### Risk assessment results produced by using the ST/DT model

**Fig. 4:**
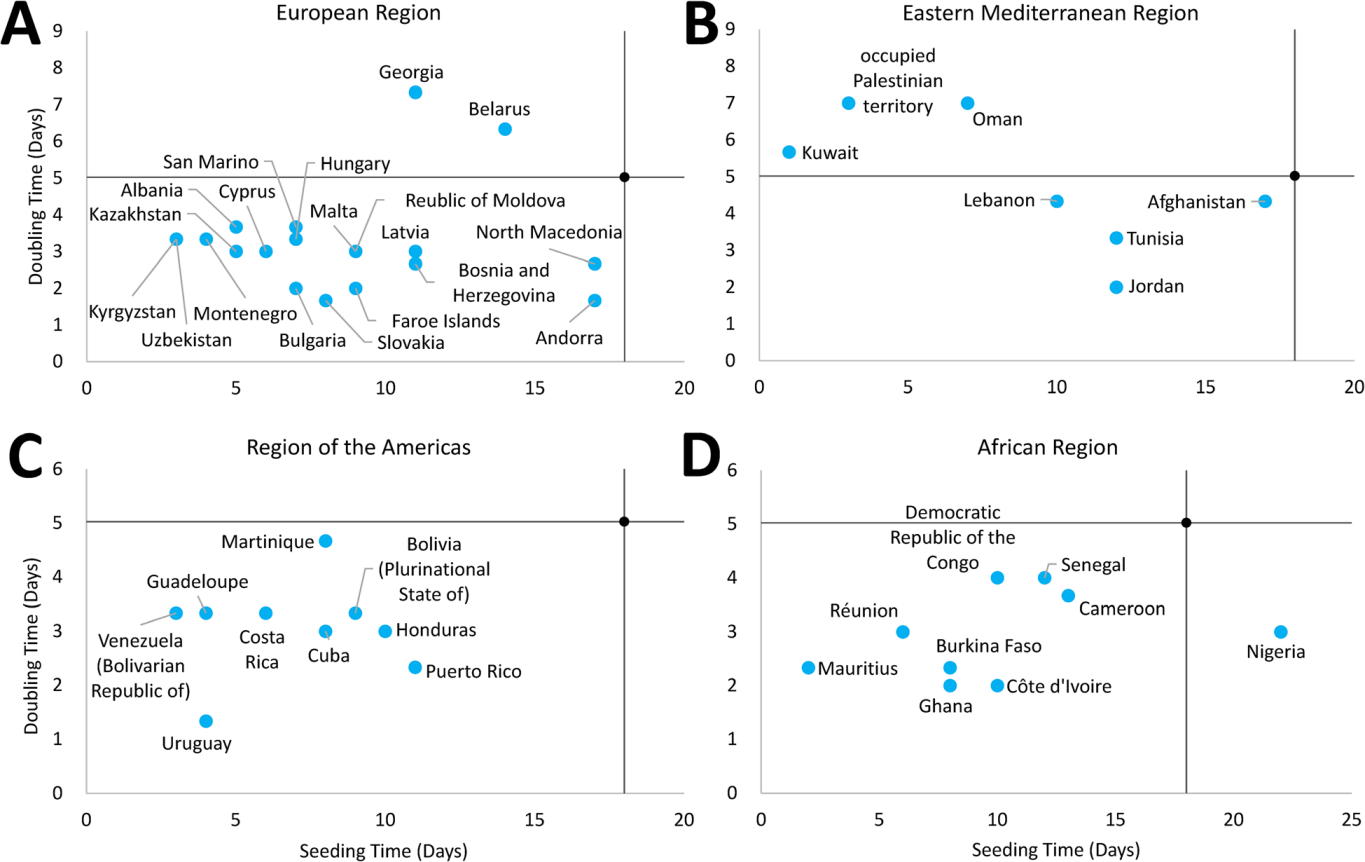
Use of the ST/DT Model to conduct risk assessments for 45 countries and territories with cumulative total numbers of cases between 100 and 500 as of 31 March 2020. **a** European Region, **b** Eastern Mediterranean Region, **c** Region of the Americas, and **d** African Region. All countries’ ST and DT placed them in the high risk quadrant except for Georgia, Belarus, Kuwait, Oman, and the occupied Palestinian territory, which were assessed as moderately low risk (long DT, but short ST) and Nigeria, which was assessed as moderately high risk (long ST, but short DT)

As shown in Fig. 4, we were able to determine the location of a sample of 45 countries on the ST/DT Model’s coordinate plane. A total of 39 countries (87%) were found to be at high risk, meaning they had short ST and short DT. Of note, Belarus and Georgia in the European Region were found to have moderately low risk (i.e., short ST, long DT) as did Kuwait, Oman, and the occupied Palestinian territory in the Eastern Mediterranean Region. The only country assessed at moderately high risk was Nigeria, in the African Region. No countries were assessed as low risk. Summarized data used to conduct the risk assessments using the ST/DT Model can be found in the Additional file 1: Table S4.

### Model validation (results of sensitivity analyses)

To validate that the changes in risk assessment over time that the model detected were meaningful, we compared these changes with official country-specific reports of epidemic features and response measures for two countries—Australia and Belarus.

Australia’s first three DTs were long, having a mean of 13 days from 1 February (12-case SN reached) to 10 March (cumulative total of 92 cases reported; Fig. 3a). During this time, national weekly epidemiological reports indicated that all known cases were linked to travel from China, Iran (Islamic Republic of), or the Diamond Princess cruise ship. The only response actions taken during this period, besides isolation of known cases, were travel restrictions to the mainland of China (13 February), Iran (29 February), and Republic of Korea (5 March) [14–18]. Australia’s second three DTs were dramatically shorter, having a mean of 3 days from 11 March (112 cumulative cases) to 19 March (681 cumulative cases; Fig. 3b). On 7 March, a total of 15 cases were determined to have no recent overseas travel history, suggesting that community spread had started [18]. Just 1 week later, 34% of cases were known to have been locally acquired [19]. Broader population-level response measures within Australia only began to be implemented on 20 March [19]. Given this context, we would expect Australia to move from an assessment of moderately low risk, to an assessment of high risk—the virus was taking hold, community transmission was becoming more common, and case counts were climbing, yet more aggressive response measures, such as social distancing, had not yet been implemented.

**Fig. 5:**
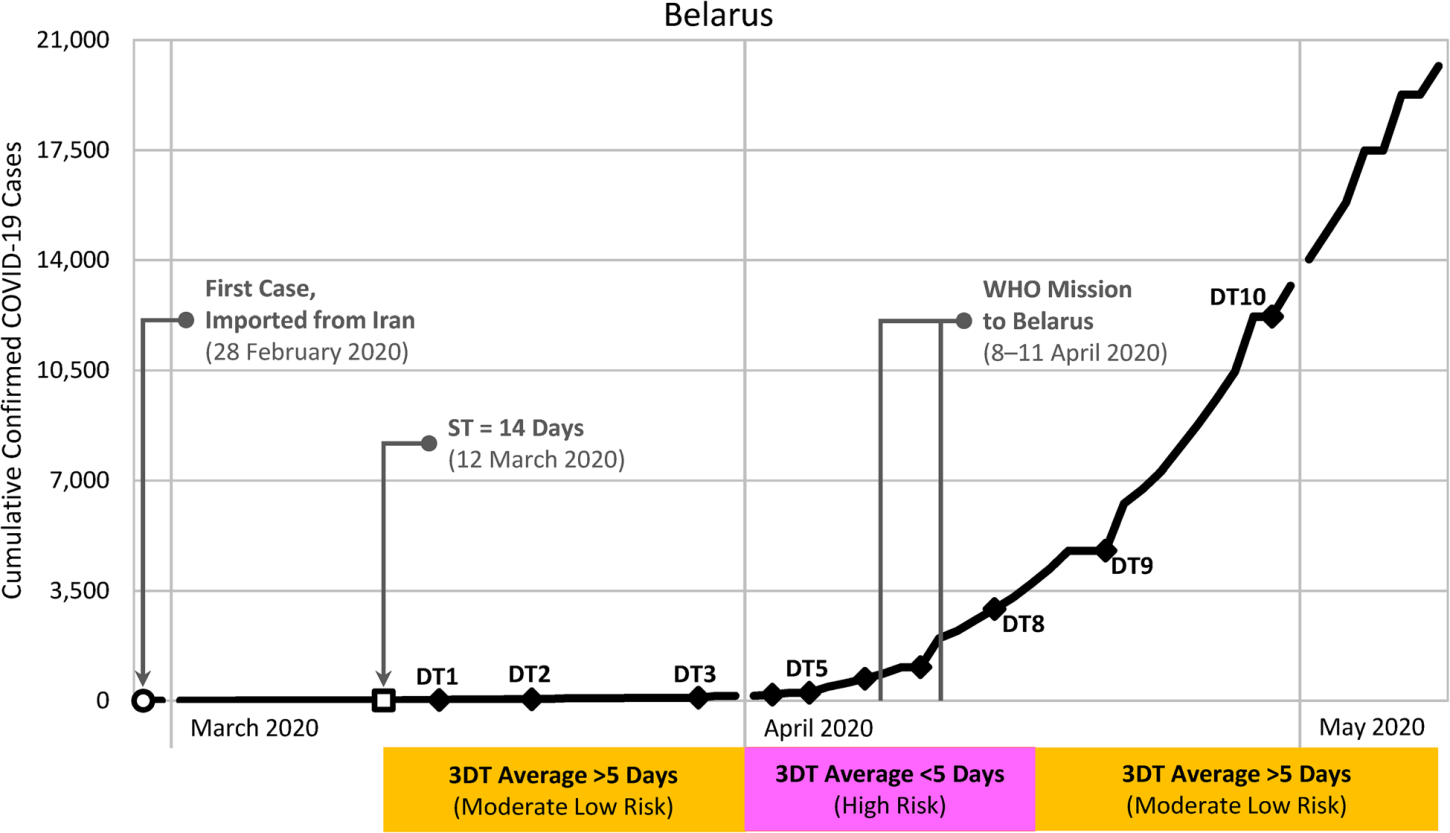
Epidemiologic curve of Belarus as an illustration of how risk assessment changes over time, 28 February 2020 to 8 May 2020. Belarus reached the SN (12 cases) on 12 March, for an ST of 14 days (below the 18-day mean ST). The average of its first 3 DTs (all DTs marked on the curve as black diamonds) was 5.7 days, placing Belarus initially in the moderately low risk (yellow) quadrant of the ST/DT Model. However, as DT began to shorten, Belarus crossed over into the ST/DT Model’s high risk (red) quadrant and the Minister of Health requested assistance from WHO. Although Belarus had been testing suspected cases; isolating confirmed cases, first level contacts, and new arrivals from COVID-19 affected countries; and partially implementing voluntary social distancing while scaling up hospital capacity, these measures had clearly not been enough. Key recommendations to Belarus by WHO included dramatic increase in mandatory social distancing (e.g., cancelling events and gatherings, implementing remote work and school, closing non-essential businesses, restricting non-essential movement), scale up of testing (i.e., expanding testing formats, ensuring quality of test kits, and strengthening entry screening), and strengthening of health system infrastructure [20]. A notable lengthening in DT has since been observed, returning Belarus to moderately low risk in the ST/DT Model

The results of using Belarus as an example country for model validation are shown in Fig. 5. The first case of COVID-19 was reported in Belarus on 28 February. Fourteen days later there were 12 cases and thus, the SN had been reached. So, ST was < 18 days and the mean of the first three DTs was > 5 days, placing Belarus originally in the moderately low risk quadrant of the ST/DT Model (Fig. 4a). However, although testing was being scaled up, cases and first level contacts were being isolated, and some screening was being done at major national entry points, social distancing was only partially implemented, and enforcement was limited [20]. Thus, DT began to shorten—the epidemic was accelerating. Rolling 3DT averages (i.e., average of DT1–3 vs DT2–4 vs. DT3–5, etc.) indicated that mean DT was declining, and Belarus crossed over into the high risk quadrant of the model. Mean DT got as low as ~ 3 days, and WHO responded to a request for assistance by visiting, conducting an assessment, and reporting on key recommendations for implementation of enhanced control measures. DT then lengthened again, exceeding 5 days, and Belarus re-entered the moderately low risk quadrant. Although its epidemic is still growing, it is not expanding at the same rapid rate as before.

## Discussion

In this study, we used publicly available COVID-19 case reporting data at the country/territory level to develop a simple model that can be used to assess the risk of an outbreak “taking off.” This ST/DT Model is intended to be iteratively used at the national level by policymakers and others to evaluate their outbreaks and the effectiveness of their response efforts. We used this model to conduct a risk assessment on outbreaks in 45 countries/territories in 4 regions that still had between 100 and 500 cases as of 31 March 2020. Our main finding was that 87% (39/45) were at high risk for their outbreak taking off and entering a rapidly growing epidemic stage. These countries needed to take immediate action to implement control measures. Although these 45 countries represent less than 10% of the global population, most are low or middle income, and therefore likely do not have the health system infrastructure capable of handling the enormous numbers of infections already being experienced by countries further along in their epidemics. Indeed, full-blown COVID-19 epidemics in these countries have the potential to produce serious humanitarian disasters.

The sensitivity analysis we conducted with the ST/DT Model verified that it could detect changes in two directions—increasing DT thereby lowering risk, and decreasing DT thereby heightening risk. These changes reflect a complex combination of factors related to the epidemic itself (e.g., transmission dynamics) and countries’ responses to the epidemic (i.e., containment and mitigation strategies and when and how well they are implemented). To validate that the changes the model detected were meaningful, we compared these changes with COVID-19 reporting from two countries. For Australia, the ST/DT model was able to detect shortening of DT and changing of risk assessment from moderately low to high risk ahead of Australia’s implementation of social distancing measures [19]. For Belarus, the ST/DT Model was able to detect shortening of DT and changing of risk assessment from moderately low to high risk when social distancing was only voluntary and partially implemented. However, after consultation with WHO, Belarus implemented more strict measures nationwide [20]. DT has since lengthened. The ST/DT Model indicated that Belarus had returned to moderately low risk. These results validate our ST/DT Model, indicating that it can produce meaningful assessments.

Despite this demonstration that our ST/DT Model for COVID-19 epidemic risk assessment can add value as decision makers across the globe appraise their epidemic response options, our model does have several limitations. Firstly, since we intentionally built the ST/DT Model to only use case reporting data, so that it would be easy, convenient, and intuitive to use for nations lacking sophisticated data collection capacity, this decision does limit the model in the sense that its output is only as good as the case report data input. These data may be biased by the factors that can influence the quality and quantity of case reporting, which will be different in each individual country. This information bias could not only affect individual countries’ risk assessments, but could also affect the median SN, mean ST, and mean DT used to structure the model. However, we attempted to mitigate this risk by taking a relatively large sample of countries in similar stages of their epidemics to determine the set point for these model variables. Secondly, the mean early epidemic DT across 20 countries was found to be 5 days, which is consistent with SARS-CoV-2 incubation period estimates,^11^ lending confidence to this model parameter. Nevertheless, as more is learned and more cases are reported mean DT, and other model parameters, will need to be updated to improve the ST/DT Model as more and better information becomes available.

## Conclusions

In summary, as COVID-19 spreads rapidly around the world, and each nation encounters it at a slightly different times and under different conditions, each government must decide when and how to respond to this very serious health threat. This decision-making process must include some evaluation of the level of risk the nation faces with respect to whether their outbreak will expand rapidly into a large epidemic. Furthermore, many countries, particularly those with weaker public health infrastructure, may only have case report data and may lack the capacity required to develop their own sophisticated modeling. Our simple, intuitive, and pragmatic ST/DT Model meets this urgent need and can serve as a tool monitor the effectiveness of response measures, promoting timely and informed decision making.

## Supplementary information

**Additional file 1: Table S1.** All countries/territories with at least one COVID-19 case as of Mar 31, 2020. **Table S2.** Raw data used for seeding number (SN), seeding time (ST), and doubling time (DT). **Table S3.** Calculation of median SN and mean ST and DT, and inputs for sensitivity analysis. **Table S4.** ST and DT values used for all countries/territories assessed for risk using the ST/DT model.

## Data Availability

The datasets analyzed during the current study are available in the WHO COVID-19 Situation Reports, https://www.who.int/emergencies/diseases/novel-coronavirus-2019/situation-reports/.
