# Supplementary material for: COVID-19 seeding time and doubling time model: an early epidemic risk assessment tool
Source: Infect Dis Poverty. 2020 Jun 23;9:76. doi: 10.1186/s40249-020-00685-4 (PMC7309203; doi:10.1186/s40249-020-00685-4)
Supplement: Supplementary file 1 — Additional file 1: Table S1. All countries/territories with at least one COVID-19 case as of Mar 31, 2020. Table S2. Raw data used for seeding number (SN), seeding time (ST), and doubling time (DT). Table S3. Calculation of median SN and mean ST and DT, and inputs for sensitivity analysis. Table S4. ST and DT values used for all countries/territories assessed for risk using the ST/DT model. [file 40249_2020_685_MOESM1_ESM.docx]

**ADDITIONAL FILE**

**COVID-19 seeding time and doubling time model:**

**an early epidemic risk assessment tool**

Lei Zhou, Jiangmei Liu, Xiaoping Dong, Jennifer M. McGoogan, Zunyou Wu

**Table of Contents**

Table S1. All countries/territories with at least one COVID-19 case as of Mar 31, 2020 page 1

Table S2. Raw data used for seeding number (SN), seeding time (ST), and doubling time (DT) page 5

Table S3. Calculation of median SN and mean ST and DT, and inputs for sensitivity analysis page 8

Table S4. ST and DT values used for all countries/territories assessed for risk using the ST/DT model page 9

**Table S1. All countries/territories with at least one COVID-19 case as of Mar 31, 2020.**

| **Country/Territory^a,b^** | **Date of First Case^c^** | **Total Cases as of Mar 31, 2020^c^** |
| --- | --- | --- |
| United States of America | Jan 24, 2020 | 187 848 |
| Italy | Jan 31, 2020 | 105 792 |
| Spain | Feb 2, 2020 | 94 417 |
| Germany | Jan 29, 2020 | 67 788 |
| France | Jan 26, 3030 | 52 128 |
| Iran (Islamic Republic of) | Feb 20, 2020 | 44 606 |
| The United Kingdom | Feb 2, 2020 | 25 150 |
| Switzerland | Feb 26, 2020 | 16 176 |
| Turkey | Mar 10, 2020 | 13 531 |
| Belgium | Feb 6, 2020 | 12 775 |
| Netherlands | Feb 27, 2020 | 12 662 |
| Republic of Korea | Jan 21, 2020 | 9887 |
| Austria | Feb 26, 2020 | 9876 |
| Canada | Jan 28, 2020 | 7708 |
| Portugal | Mar 4, 2020 | 7443 |
| Israel | Feb 22, 2020 | 4831 |
| Brazil | Feb 26, 2020 | 4661 |
| Norway | Feb 26, 2020 | 4592 |
| Australia | Jan 26, 2020 | 4559 |
| Sweden | Feb 2, 2020 | 4435 |
| Czechia | Mar 2, 2020 | 3002 |
| Denmark | Feb 26, 2020 | 2994 |
| Ireland | Feb 29, 2020 | 2910 |
| Malaysia | Jan 27, 2020 | 2766 |
| Chile | Mar 2, 2020 | 2738 |
| Russian Federation | Feb 2, 2020 | 2337 |
| Romania | Feb 26, 2020 | 2245 |
| Poland | Mar 3, 2020 | 2215 |
| Japan | Jan 7, 2020 | 2212 |
| Luxembourg | Feb 29, 2020 | 2178 |
| Philippines | Jan 31, 2020 | 2084 |
| Ecuador | Feb 26, 2020 | 1966 |
| Pakistan | Feb 26, 2020 | 1865 |
| Thailand | Jan 22, 2020 | 1651 |
| Saudi Arabia | Mar 3, 2020 | 1563 |
| Indonesia | Feb 29, 2020 | 1528 |
| Finland | Jan 31, 2020 | 1418 |
| South Africa | Mar 4, 2020 | 1326 |
| Greece | Feb 26, 2020 | 1314 |
| India | Jan 31, 2020 | 1251 |
| Iceland | Feb 27, 2020 | 1135 |
| Mexico | Feb 27, 2020 | 1094 |
| Panama | Mar 10, 2020 | 1075 |
| Argentina | Mar 2, 2020 | 966 |
| Peru | Mar 5, 2020 | 950 |
| Singapore | Jan 25, 2020 | 926 |
| Dominican Republic | Mar 1, 2020 | 901 |
| Serbia | Mar 5, 2020 | 900 |
| Croatia | Feb 26, 2020 | 867 |
| Slovenia | Mar 4, 2020 | 802 |
| Colombia | Mar 5, 2020 | 798 |
| Estonia | Feb 26, 2020 | 745 |
| Iraq | Feb 26, 2020 | 694 |
| Qatar | Feb 29, 2020 | 693 |
| United Arab Emirates | Jan 30, 2020 | 664 |
| Egypt | Feb 16, 2020 | 656 |
| New Zealand | Feb 27, 2020 | 647 |
| Algeria | Feb 26, 2020 | 584 |
| Morocco | Mar 2, 2020 | 574 |
| Bahrain | Feb 26, 2020 | 567 |
| Ukraine | Mar 3, 2020 | 549 |
| Lithuania | Feb 27, 2020 | 533 |
| Armenia | Feb 29, 2020 | 532 |
| Hungary | Mar 4, 2020 | 492 |
| Lebanon | Feb 22, 2020 | 463 |
| Bosnia and Herzegovina | Mar 4, 2020 | 411 |
| Latvia | Mar 1, 2020 | 398 |
| Bulgaria | Mar 7, 2020 | 379 |
| Andorra | Feb 29, 2020 | 370 |
| Slovakia | Mar 5, 2020 | 363 |
| Tunisia | Mar 2, 2020 | 362 |
| Republic of Moldova | Mar 6, 2020 | 353 |
| Kazakhstan | Mar 12, 2020 | 336 |
| Costa Rica | Mar 5, 2020 | 330 |
| Uruguay | Mar 13, 2020 | 320 |
| Azerbaijan | Feb 27, 2020 | 298 |
| Kuwait | Feb 25, 2020 | 289 |
| North Macedonia | Feb 26, 2020 | 285 |
| Jordan | Mar 4, 2020 | 268 |
| Burkina Faso | Mar 9, 2020 | 246 |
| Albania | Mar 8, 2020 | 243 |
| Cyprus | Mar 9, 2020 | 230 |
| San Marino | Feb 27, 2020 | 230 |
| Réunion | Mar 3, 2020 | 207 |
| Viet Nam | Jan 25, 2020 | 207 |
| Cameroon | Mar 6, 2020 | 193 |
| Oman | Feb 26, 2020 | 192 |
| Senegal | Mar 2, 2020 | 175 |
| Afghanistan | Feb 26, 2020 | 174 |
| Puerto Rico | Mar 13, 2020 | 174 |
| Cuba | Mar 12, 2020 | 170 |
| Malta | Mar 5, 2020 | 169 |
| Côte d’Ivoire | Mar 11, 2020 | 168 |
| Faroe Islands | Mar 9, 2020 | 168 |
| Uzbekistan | Mar 15, 2020 | 167 |
| Belarus | Feb 27, 2020 | 152 |
| Ghana | Mar 12, 2020 | 152 |
| Mauritius | Mar 19, 2020 | 143 |
| Sri Lanka | Jan 29, 2020 | 142 |
| Honduras | Mar 10, 2020 | 141 |
| Nigeria | Feb 27, 2020 | 135 |
| Venezuela (Bolivarian Republic of) | Mar 13, 2020 | 135 |
| Brunei Darussalam | Mar 9, 2020 | 129 |
| occupied Palestinian territory | Mar 4, 2020 | 117 |
| Martinique | Mar 8, 2020 | 111 |
| Georgia | Feb 26, 2020 | 110 |
| Cambodia | Jan 29, 2020 | 110 |
| Bolivia (Plurinational State of) | Mar 11, 2020 | 107 |
| Kyrgyzstan | Mar 18, 2020 | 107 |
| Guadeloupe | Mar 14, 2020 | 106 |
| Montenegro | Mar 17, 2020 | 105 |
| Democratic Republic of the Congo | Mar 9, 2020 | 98 |
| Trinidad and Tobago | Mar 12, 2020 | 85 |
| Mayotte | Mar 15, 2020 | 82 |
| Guam | Mar 17, 2020 | 73 |
| Rwanda | Mar 16, 2020 | 70 |
| Gibraltar | Mar 5, 2020 | 65 |
| Paraguay | Mar 6, 2020 | 65 |
| Jersey | Mar 13, 2020 | 63 |
| Liechtenstein | Mar 5, 2020 | 62 |
| Kenya | Mar 12, 2020 | 59 |
| Bangladesh | Mar 7, 2020 | 51 |
| Aruba | Mar 13, 2020 | 50 |
| Monaco | Feb 27, 2020 | 49 |
| Guernsey | Mar 10, 2020 | 48 |
| Madagascar | Mar 20, 2020 | 46 |
| French Guiana | Mar 8, 2020 | 43 |
| Guatemala | Mar 13, 2020 | 36 |
| Jamaica | Mar 10, 2020 | 36 |
| French Polynesia | Mar 12, 2020 | 36 |
| Zambia | Mar 17, 2020 | 36 |
| Togo | Mar 5, 2020 | 34 |
| Barbados | Mar 18, 2020 | 33 |
| Uganda | Mar 21, 2020 | 33 |
| Isle of Man | Mar 25, 2020 | 32 |
| United States Virgin Islands | Mar 17, 2020 | 30 |
| Mali | Mar 25, 2020 | 28 |
| Djibouti | Mar 17, 2020 | 25 |
| Ethiopia | Mar 12, 2020 | 25 |
| El Salvador | Mar 18, 2020 | 24 |
| Bermuda | Mar 25, 2020 | 22 |
| Guyana | Mar 12, 2020 | 20 |
| Congo | Mar 15, 2020 | 19 |
| United Republic of Tanzania | Mar 18, 2020 | 19 |
| Maldives | Mar 6, 2020 | 18 |
| Niger | Mar 19, 2020 | 18 |
| Gabon | Mar 12, 2020 | 16 |
| Guinea | Mar 12, 2020 | 16 |
| Eritrea | Mar 21, 2020 | 15 |
| Haiti | Mar 19, 2020 | 15 |
| Myanmar | Mar 23, 2020 | 15 |
| New Caledonia | Mar 25, 2020 | 15 |
| Bahamas | Mar 15, 2020 | 14 |
| Equatorial Guinea | Mar 13, 2020 | 14 |
| Saint Martin | Mar 7, 2020 | 12 |
| Mongolia | Mar 10, 2020 | 12 |
| Dominica | Mar 23, 2020 | 11 |
| Namibia | Mar 13, 2020 | 11 |
| Greenland | Mar 17, 2020 | 10 |
| Syrian Arab Republic | Mar 22, 2020 | 10 |
| Grenada | Mar 23, 2020 | 9 |
| Saint Lucia | Mar 17, 2020 | 9 |
| Swaziland | Mar 16, 2020 | 9 |
| Cayman Islands | Mar 14, 2020 | 8 |
| Saint Kitts and Nevis | Mar 25, 2020 | 8 |
| Lao People’s Democratic Republic | Mar 23, 2020 | 8 |
| Libya | Mar 24, 2020 | 8 |
| Mozambique | Mar 23, 2020 | 8 |
| Suriname | Mar 14, 2020 | 8 |
| Seychelles | Mar 14, 2020 | 8 |
| Zimbabwe | Mar 20, 2020 | 8 |
| Angola | Mar 20, 2020 | 7 |
| Netherlands Antilles | Mar 15, 2020 | 7 |
| Antigua and Barbuda | Mar 13, 2020 | 7 |
| Sudan | Mar 12, 2020 | 7 |
| Benin | Mar 16, 2020 | 6 |
| Central African Republic | Mar 14, 2020 | 6 |
| Cabo Verde | Mar 22, 2020 | 6 |
| Holy See | Mar 5, 2020 | 6 |
| Fiji | Mar 18, 2020 | 5 |
| Mauritania | Mar 13, 2020 | 5 |
| Monserrat | Mar 19, 2020 | 5 |
| Nepal | Jan 26, 2020 | 5 |
| Chad | Mar 19, 2020 | 5 |
| Bhutan | Mar 4, 2020 | 4 |
| Gambia | Mar 17, 2020 | 4 |
| Nicaragua | Mar 20, 2020 | 4 |
| Turks and Caicos Islands | Mar 27, 2020 | 4 |
| Saint Barthélemy | Mar 7, 2020 | 3 |
| Botswana | Mar 30, 2020 | 3 |
| Liberia | Mar 18, 2020 | 3 |
| Somalia | Mar 18, 2020 | 3 |
| Anguilla | Mar 27, 2020 | 2 |
| Burundi | Mar 31, 2020 | 2 |
| Belize | Mar 23, 2020 | 2 |
| Guinea-Bissau | Mar 25, 2020 | 2 |
| Northern Mariana Islands (Commonwealth of the) | Mar 29, 2020 | 2 |
| British Virgin Islands | Mar 27, 2020 | 2 |
| Paupa New Guinea | Mar 20, 2020 | 1 |
| Sierra Leone | Mar 30, 2020 | 1 |
| Timor-Leste | Mar 20, 2020 | 1 |
| Saint Vincent and the Grenadines | Mar 13, 2020 | 1 |

^a^Naming of countries and territories is in accordance with World Health Organization COVID-19 daily situation reports.

^b^China was excluded since its epidemic curve was much further ahead compared to all other countries/territories with very few cases still being reported by Mar 31, 2020.

^c^Data extracted from World Health Organization COVID-19 daily situation reports.

**Table S2. Raw data used for seeding number (SN), seeding time (ST), and doubling time (DT).** Colored cells and colored numerical values are meant to guide inputs into Table A3 below.

|  | **Countries^a,b,c^** | | | | | | | | | | | | | | | | | | | | | | | | | | | | | |
| --- | --- | --- | --- | --- | --- | --- | --- | --- | --- | --- | --- | --- | --- | --- | --- | --- | --- | --- | --- | --- | --- | --- | --- | --- | --- | --- | --- | --- | --- | --- |
| **Date** | **Australia** | **Austria** | **Belgium** | **Cambodia** | **Canada** | **Egypt** | **Finland** | **France** | **Germany** | **India** | **Iran (Islamic Republic of)** | **Italy** | **Japan** | **Republic of Korea** | **Malaysia** | **Netherlands** | **Philippines** | **Portugal** | **Russian Federation** | **Singapore** | **Spain** | **Sri Lanka** | **Sweden** | **Switzerland** | **Thailand** | **Turkey** | **United Arab Emirates** | **The United Kingdom** | **United States of America** | **Viet Nam** |
| Jan 7 |  |  |  |  |  |  |  |  |  |  |  |  | 1 |  |  |  |  |  |  |  |  |  |  |  |  |  |  |  |  |  |
| Jan 8 |  |  |  |  |  |  |  |  |  |  |  |  | 1 |  |  |  |  |  |  |  |  |  |  |  |  |  |  |  |  |  |
| Jan 9 |  |  |  |  |  |  |  |  |  |  |  |  | 1 |  |  |  |  |  |  |  |  |  |  |  |  |  |  |  |  |  |
| Jan 10 |  |  |  |  |  |  |  |  |  |  |  |  | 1 |  |  |  |  |  |  |  |  |  |  |  |  |  |  |  |  |  |
| Jan 11 |  |  |  |  |  |  |  |  |  |  |  |  | 1 |  |  |  |  |  |  |  |  |  |  |  |  |  |  |  |  |  |
| Jan 12 |  |  |  |  |  |  |  |  |  |  |  |  | 1 |  |  |  |  |  |  |  |  |  |  |  |  |  |  |  |  |  |
| Jan 13 |  |  |  |  |  |  |  |  |  |  |  |  | 1 |  |  |  |  |  |  |  |  |  |  |  |  |  |  |  |  |  |
| Jan 14 |  |  |  |  |  |  |  |  |  |  |  |  | 1 |  |  |  |  |  |  |  |  |  |  |  |  |  |  |  |  |  |
| Jan 15 |  |  |  |  |  |  |  |  |  |  |  |  | 1 |  |  |  |  |  |  |  |  |  |  |  |  |  |  |  |  |  |
| Jan 16 |  |  |  |  |  |  |  |  |  |  |  |  | 1 |  |  |  |  |  |  |  |  |  |  |  |  |  |  |  |  |  |
| Jan 17 |  |  |  |  |  |  |  |  |  |  |  |  | 1 |  |  |  |  |  |  |  |  |  |  |  |  |  |  |  |  |  |
| Jan 18 |  |  |  |  |  |  |  |  |  |  |  |  | 1 |  |  |  |  |  |  |  |  |  |  |  |  |  |  |  |  |  |
| Jan 19 |  |  |  |  |  |  |  |  |  |  |  |  | 1 |  |  |  |  |  |  |  |  |  |  |  |  |  |  |  |  |  |
| Jan 20 |  |  |  |  |  |  |  |  |  |  | 2 |  | 1 |  |  |  |  |  |  |  |  |  |  |  |  |  |  |  |  |  |
| Jan 21 |  |  |  |  |  |  |  |  |  |  | **5** |  | 1 | 1 |  |  |  |  |  |  |  |  |  |  |  |  |  |  |  |  |
| Jan 22 |  |  |  |  |  |  |  |  |  |  | **18** |  | 1 | 1 |  |  |  |  |  |  |  |  |  |  | 2 |  |  |  |  |  |
| Jan 23 |  |  |  |  |  |  |  |  |  |  | 28 |  | 1 | 1 |  |  |  |  |  |  |  |  |  |  | 4 |  |  |  |  |  |
| Jan 24 |  |  |  |  |  |  |  |  |  |  | **43** |  | 2 | 2 |  |  |  |  |  |  |  |  |  |  | 4 |  |  |  | 1 |  |
| Jan 25 |  |  |  |  |  |  |  |  |  |  | 61 |  | 3 | 2 |  |  |  |  |  | 3 |  |  |  |  | 4 |  |  |  | 2 | 2 |
| Jan 26 | 4 |  |  |  |  |  |  | 3 |  |  | **95** |  | 3 | 3 |  |  |  |  |  | 4 |  |  |  |  | 5 |  |  |  | 2 | 2 |
| Jan 27 | 4 |  |  |  |  |  |  | 3 |  |  | **141** |  | 3 | 4 | 4 |  |  |  |  | 4 |  |  |  |  | **5** |  |  |  | 5 | 2 |
| Jan 28 | 5 |  |  |  | 2 |  |  | 3 |  |  | **245** |  | 3 | 4 | 4 | 1 |  |  |  | 7 |  |  |  |  | 14 |  |  |  | 5 | 2 |
| Jan 29 | 7 |  |  | 1 | 3 |  |  | 4 | 4 |  | 388 |  | 3 | 4 | 4 | 2 |  |  |  | 7 |  |  |  |  | 14 |  |  |  | 5 | 2 |
| Jan 30 | 7 |  |  | 1 | 3 |  |  | 5 | 4 |  | **593** |  | 3 | 6 | 7 | **7** |  |  |  | **10** |  | 1 |  |  | 14 |  | 4 |  | 5 | 2 |
| Jan 31 | 9 |  |  | 1 | 3 |  | 1 | 6 | 5 | 1 | 978 | 2 | **3** | 11 | 8 | **13** | 1 |  |  | 13 |  | 1 |  |  | **14** |  | 4 |  | 6 | 5 |
| Feb 1 | **12** |  |  | 1 | 4 |  | 1 | 6 | 7 | 1 | … | 2 | **20** | 12 | 8 | **18** | 1 |  |  | 16 |  | 1 |  |  | 19 |  | 4 |  | 7 | 6 |
| Feb 2 | 12 |  |  | 1 | 4 |  | 1 | 6 | 8 | 2 |  | 2 | 20 | 15 | 8 | 28 | 2 |  | 2 | 18 | 1 | 1 | 1 |  | 19 |  | 5 | 2 | 8 | 7 |
| Feb 3 | 12 |  |  | 1 | 4 |  | 1 | 6 | 10 | 3 |  | 2 | 20 | 16 | 8 | **38** | 2 |  | 2 | 18 | 1 | 1 | 1 |  | 19 |  | 5 | 2 | 11 | 8 |
| Feb 4 | 12 |  |  | 1 | 4 |  | 1 | 6 | 12 | 3 |  | 2 | **20** | 16 | 10 | **82** | 2 |  | 2 | 18 | 1 | 1 | 1 |  | **19** |  | 5 | 2 | 11 | 9 |
| Feb 5 | 13 |  |  | 1 | 5 |  | 1 | 6 | 12 | 3 |  | 2 | 23 | 19 | 10 | 128 | 3 |  | 2 | **24** | 1 | 1 | 1 |  | 25 |  | 5 | 2 | 11 | 10 |
| Feb 6 | 14 |  | 1 | 1 | 5 |  | 1 | 6 | 12 | 3 |  | 3 | 25 | 23 | **12** | **188** | 3 |  | 2 | 28 | 1 | 1 | 1 |  | 25 |  | 5 | 2 | 12 | 10 |
| Feb 7 | 15 |  | 1 | 1 | 7 |  | 1 | 6 | 13 | 3 |  | 3 | 25 | 24 | 14 | 265 | 3 |  | 2 | 30 | 1 | 1 | 1 |  | 25 |  | 5 | 3 | 12 | **12** |
| Feb 8 | 15 |  | 1 | 1 | 7 |  | 1 | 6 | 14 | 3 |  | 3 | 25 | 24 | 15 | 321 | 3 |  | 2 | 33 | 1 | 1 | 1 |  | 32 |  | 7 | 3 | 12 | 13 |
| Feb 9 | 15 |  | 1 | 1 | 7 |  | 1 | 11 | 14 | 3 |  | 3 | 26 | 25 | 17 | **382** | 3 |  | 2 | 40 | 1 | 1 | 1 |  | 32 |  | 7 | 3 | 12 | 14 |
| Feb 10 | 15 |  | 1 | 1 | 7 |  | 1 | 11 | 14 | 3 |  | 3 | 26 | 27 | 18 | 503 | 3 |  | 2 | 43 | 2 | 1 | 1 |  | 32 |  | 7 | 4 | 12 | 14 |
| Feb 11 | 15 |  | 1 | 1 | 7 |  | 1 | 11 | 14 | 3 |  | 3 | 26 | 28 | 18 | **614** | 3 |  | 2 | 45 | 2 | 1 | 1 |  | 33 |  | 8 | 8 | 13 | 15 |
| Feb 12 | 15 |  | 1 | 1 | 7 |  | 1 | 11 | 16 | 3 |  | 3 | 28 | 28 | 18 | 804 | 3 |  | 2 | **47** | 2 | 1 | 1 |  | 33 |  | 8 | 8 | 13 | 15 |
| Feb 13 | 15 |  | 1 | 1 | 7 |  | 1 | 11 | 16 | 3 |  | 3 | 29 | 28 | 18 | … | 3 |  | 2 | 50 | 2 | 1 | 1 |  | 33 |  | 8 | 9 | 14 | 16 |
| Feb 14 | 15 |  | 1 | 1 | 7 |  | 1 | 11 | 16 | 3 |  | 3 | 33 | 28 | 19 |  | 3 |  | 2 | 58 | 2 | 1 | 1 |  | 33 |  | 8 | 9 | 15 | 16 |
| Feb 15 | 15 |  | 1 | 1 | 7 |  | 1 | 11 | 16 | 3 |  | 3 | **41** | 28 | 21 |  | 3 |  | 2 | 67 | 2 | 1 | 1 |  | 34 |  | 8 | 9 | 15 | 16 |
| Feb 16 | 15 |  | 1 | 1 | 7 | 1 | 1 | 12 | 16 | 3 |  | 3 | 53 | 28 | 22 |  | 3 |  | 2 | 72 | 2 | 1 | 1 |  | 34 |  | 8 | 9 | 15 | 16 |
| Feb 17 | 15 |  | 1 | 1 | 7 | 1 | 1 | 12 | 16 | 3 |  | 3 | 59 | **28** | 22 |  | 3 |  | 2 | 75 | 2 | 1 | 1 |  | 35 |  | 9 | 9 | 15 | 16 |
| Feb 18 | 15 |  | 1 | 1 | 8 | 1 | 1 | 12 | 16 | 3 |  | 3 | 65 | 31 | 22 |  | 3 |  | 2 | 77 | 2 | 1 | 1 |  | 35 |  | 9 | 9 | 15 | 16 |
| Feb 19 | 15 |  | 1 | 1 | 8 | 1 | 1 | 12 | 16 | 3 |  | 3 | 73 | 51 | 22 |  | 3 |  | 2 | 81 | 2 | 1 | 1 |  | 35 |  | 9 | 9 | 15 | 16 |
| Feb 20 | 15 |  | 1 | 1 | 8 | 1 | 1 | 12 | 16 | 3 |  | **4** | 85 | 104 | 22 |  | 3 |  | 2 | 84 | 2 | 1 | 1 |  | 35 |  | 9 | 9 | 15 | 16 |
| Feb 21 | **17** |  | 1 | 1 | 8 | 1 | 1 | 12 | 16 | 3 |  | **20** | **93** | 204 | 22 |  | 3 |  | 2 | 85 | 2 | 1 | 1 |  | 35 |  | 9 | 9 | **15** | 16 |
| Feb 22 | 21 |  | 1 | 1 | 8 | 1 | 1 | 12 | 16 | 3 |  | **79** | 105 | 433 | 22 |  | 3 |  | 2 | 86 | 2 | 1 | 1 |  | 35 |  | **11** | 9 | 35 | 16 |
| Feb 23 | 22 |  | 1 | 1 | 9 | 1 | 1 | 12 | 16 | 3 |  | **132** | 132 | 602 | 22 |  | 3 |  | 2 | 89 | 2 | 1 | 1 |  | 35 |  | 13 | 9 | 35 | 16 |
| Feb 24 | 22 |  | 1 | 1 | 9 | 1 | 1 | 12 | 16 | 3 |  | 229 | 144 | 833 | 22 |  | 3 |  | 2 | 89 | 2 | 1 | 1 |  | 35 |  | 13 | 9 | 35 | 16 |
| Feb 25 | 22 |  | 1 | 1 | 10 | 1 | 1 | 12 | 16 | 3 |  | 283 | 157 | … | 22 |  | 3 |  | 2 | 90 | 2 | 1 | 1 |  | 37 |  | 13 | 13 | 53 | 16 |
| Feb 26 | 23 | 2 | 1 | 1 | 10 | 1 | 1 | **12** | **18** | 3 |  | **322** | 164 |  | 22 |  | 3 |  | 2 | 91 | 2 | 1 | 1 | 1 | 40 |  | 13 | 13 | 53 | 16 |
| Feb 27 | 23 | 2 | 1 | 1 | 11 | 1 | 2 | 18 | 21 | 3 |  | 400 | **186** |  | **22** |  | 3 |  | 2 | 93 | **12** | 1 | 2 | 1 | 40 |  | **13** | **13** | 59 | 16 |
| Feb 28 | 23 | 4 | 1 | 1 | **11** | 1 | 2 | 38 | 26 | 3 |  | **650** | 210 |  | **24** |  | 3 |  | 2 | **96** | 25 | 1 | 7 | 6 | 40 |  | 19 | 16 | 59 | 16 |
| Feb 29 | **24** | 5 | 1 | 1 | 14 | 1 | 2 | 57 | 57 | 3 |  | 888 | 230 |  | 24 |  | 3 |  | 2 | 98 | 32 | 1 | **12** | **10** | 42 |  | 19 | 20 | 62 | 16 |
| Mar 1 | 25 | 10 | 1 | 1 | 19 | 1 | 2 | 100 | 57 | 3 |  | … | 239 |  | 24 |  | 3 |  | 2 | 102 | 45 | 1 | 13 | **18** | 42 |  | 19 | 23 | 62 | 16 |
| Mar 2 | 27 | **10** | 1 | 1 | 19 | 2 | 6 | 100 | 129 | 3 |  |  | 254 |  | 24 |  | 3 |  | 2 | 106 | **45** | 1 | 14 | **24** | 42 |  | 21 | 36 | 62 | 16 |
| Mar 3 | 33 | 18 | 8 | 1 | 27 | 2 | 7 | 191 | 157 | 5 |  |  | 268 |  | **36** |  | 3 |  | 3 | 108 | 114 | 1 | 15 | 30 | 43 |  | **21** | 39 | 64 | 16 |
| Mar 4 | **43** | **24** | **8** | 1 | 30 | 2 | 7 | 212 | 196 | **6** |  |  | 284 |  | 50 |  | 3 | 2 | 3 | 110 | **151** | 1 | **24** | **37** | 43 |  | 27 | 51 | 108 | 16 |
| Mar 5 | 57 | 37 | **23** | 1 | 30 | 2 | 7 | 282 | 262 | 29 |  |  | 317 |  | 50 |  | 3 | 7 | 3 | 110 | 198 | 1 | **35** | 56 | 47 |  | 27 | 89 | 129 | 16 |
| Mar 6 | 57 | **47** | **50** | 1 | 45 | 2 | **12** | 305 | 534 | 30 |  |  | **349** |  | 55 |  | 5 | 9 | 4 | 117 | 257 | 1 | **61** | **86** | 47 |  | 27 | 118 | 148 | 16 |
| Mar 7 | 62 | **66** | 109 | 1 | 51 | **3** | 19 | 438 | 639 | 31 |  |  | 408 |  | 83 |  | 5 | **13** | 7 | 130 | **374** | 1 | 137 | 209 | **48** |  | 45 | 167 | 213 | **17** |
| Mar 8 | 74 | 104 | **169** | 2 | 57 | 48 | **19** | 579 | 795 | 34 |  |  | 455 |  | 93 |  | 6 | 21 | 7 | 138 | 430 | 1 | **161** | 264 | 50 |  | 45 | 210 | 213 | 30 |
| Mar 9 | 77 | 112 | 200 | 2 | 62 | 55 | 30 | 811 | … | 43 |  |  | 488 |  | **93** |  | **10** | 30 | 7 | 150 | **589** | 1 | 203 | **332** | 50 |  | **45** | 277 | 472 | 30 |
| Mar 10 | **92** | 131 | 239 | 2 | 77 | 59 | 40 | … |  | **44** |  |  | 514 |  | 117 |  | 33 | 41 | 7 | 160 | … | 1 | 248 | 491 | 53 |  | 59 | 323 | 696 | 31 |
| Mar 11 | 112 | **182** | 267 | 3 | 93 | 59 | 40 |  |  | 60 |  |  | 568 |  | 129 |  | **35** | 41 | **7** | 166 |  | 1 | **326** | 491 | 59 | 1 | 74 | 373 | 987 | 35 |
| Mar 12 | 122 | 302 | 314 | 3 | 93 | 67 | **40** |  |  | 73 |  |  | 620 |  | 129 |  | 52 | 41 | **20** | 178 |  | 2 | 461 | **645** | 70 | 1 | 74 | 460 | … | 39 |
| Mar 13 | **140** | **361** | **314** | 5 | 138 | 67 | 109 |  |  | 74 |  |  | 675 |  | **129** |  | 64 | 112 | 45 | **187** |  | 3 | **620** | 858 | 75 | 1 | 85 | 594 |  | 39 |
| Mar 14 | 197 | **504** | 599 | 7 | 198 | 93 | **109** |  |  | **83** |  |  | **716** |  | 197 |  | **64** | 169 | **47** | 200 |  | 6 | 775 | … | **82** | 5 | 85 | 802 |  | **48** |
| Mar 15 | 249 | 800 | **689** | **7** | 244 | 110 | 225 |  |  | 107 |  |  | 823 |  | **238** |  | 111 | 245 | 59 | 212 |  | **11** | … |  | 114 | **6** | **85** | … |  | 53 |
| Mar 16 | 298 | … | … | 12 | 339 | 126 | 244 |  |  | 113 |  |  | … |  | 553 |  | 140 | 331 | 64 | 243 |  | 19 |  |  | 114 | **18** | 98 |  |  | 57 |
| Mar 17 | **375** |  |  | 24 | 424 | 166 | 272 |  |  | 137 |  |  |  |  | **566** |  | **187** | 642 | **93** | 243 |  | 29 |  |  | **147** | **47** | 98 |  |  | 61 |
| Mar 18 | 565 |  |  | 35 | 598 | 196 | 359 |  |  | 152 |  |  |  |  | 790 |  | 202 | 785 | **147** | 313 |  | 50 |  |  | 212 | 98 | 113 |  |  | 68 |
| Mar 19 | **681** |  |  | 37 | 727 | 210 | **359** |  |  | **176** |  |  |  |  | … |  | 217 | … | 199 | **345** |  | 57 |  |  | 272 | **191** | 140 |  |  | 76 |
| Mar 20 | 877 |  |  | 47 | 873 | 256 | 400 |  |  | 223 |  |  |  |  |  |  | 230 |  | 253 | 385 |  | 71 |  |  | **322** | **359** | 140 |  |  | 85 |
| Mar 21 | … |  |  | 51 | … | 285 | 521 |  |  | 294 |  |  |  |  |  |  | 307 |  | 306 | 432 |  | 77 |  |  | 411 | **670** | 153 |  |  | **94** |
| Mar 22 |  |  |  | 53 |  | 294 | 626 |  |  | **341** |  |  |  |  |  |  | **380** |  | **367** | 455 |  | 81 |  |  | 599 | 947 | **153** |  |  | 106 |
| Mar 23 |  |  |  | 84 |  | 327 | **700** |  |  | 425 |  |  |  |  |  |  | 462 |  | 438 | 509 |  | 92 |  |  | **721** | … | 198 |  |  | 123 |
| Mar 24 |  |  |  | 87 |  | 366 | 792 |  |  | 519 |  |  |  |  |  |  | 552 |  | 498 | 558 |  | 100 |  |  | 827 |  | 248 |  |  | 132 |
| Mar 25 |  |  |  | 93 |  | 442 | … |  |  | 606 |  |  |  |  |  |  | 636 |  | **658** | 631 |  | 102 |  |  | … |  | 333 |  |  | 134 |
| Mar 26 |  |  |  | 96 |  | 456 |  |  |  | **681** |  |  |  |  |  |  | **707** |  | 840 | 707 |  | 102 |  |  |  |  | **333** |  |  | 153 |
| Mar 27 |  |  |  | 99 |  | 495 |  |  |  | 843 |  |  |  |  |  |  | 803 |  | … | **732** |  | 106 |  |  |  |  | 405 |  |  | 163 |
| Mar 28 |  |  |  | 99 |  | 536 |  |  |  |  |  |  |  |  |  |  |  |  |  | 802 |  | 110 |  |  |  |  | 468 |  |  | 174 |
| Mar 29 |  |  |  | 103 |  | 576 |  |  |  |  |  |  |  |  |  |  |  |  |  |  |  | 115 |  |  |  |  | 570 |  |  | **188** |
| Mar 30 |  |  |  | 107 |  | 609 |  |  |  |  |  |  |  |  |  |  |  |  |  |  |  | 122 |  |  |  |  | 611 |  |  | 194 |
| Mar 31 |  |  |  | 109 |  | 656 |  |  |  |  |  |  |  |  |  |  |  |  |  |  |  | 142 |  |  |  |  | **664** |  |  | 207 |

^a^Naming of countries and territories is in accordance with World Health Organization COVID-19 daily situation reports.

^b^Data extracted from World Health Organization COVID-19 daily situation reports.

^c^SN was determined using the epidemiologic curves (plotted as time in days on the x-axis versus cumulative total number of cases on the y-axis) for each country/territory. These were independently assessed by two authors who each selected the date on which each epidemic curve appeared to “take-off.” The SN was the cumulative total number of cases on the day prior to this “take-off” day. This SN is highlighted in yellow.

**Table S3. Calculation of median SN and mean ST and DT, and inputs for sensitivity analysis.**

| **Country^a^** | **Actual SN,**  **cases^b^** | **ST, days (1→12 cases)^c^** | **Early Epidemic (1st Three Doubling Intervals)** | | | | **Later Epidemic (2nd Three Doubling Intervals)** | | | | **Change in Mean DT, days^f^** |
| --- | --- | --- | --- | --- | --- | --- | --- | --- | --- | --- | --- |
|  |  |  | **DT1, days (12→24 cases)^d^** | **DT2, days (24→48 cases)^d^** | **DT3, days**  **(48→96 cases)^d^** | **Mean DT, days** | **DT4, days**  **(96→192 cases)^e^** | **DT5, days**  **(192→384 cases)^e^** | **DT6, days**  **(384→768 cases)^e^** | **Mean DT, days** |  |
| Australia | 17 | 7 | 29 | 4 | 6 | 13.00 | 3 | 4 | 2 | 3.00 | –10.0 |
| Austria | 10 | 6 | 2 | 2 | 1 | 1.67 | 4 | 2 | 1 | 2.33 | +0.7 |
| Belgium | 23 | 28 | 1 | 0 | 1 | 0.67 | 2 | 5 | 2 | 3.00 | +2.3 |
| Cambodia | 7 | – | – | – | – | – | – | – | – | – | – |
| Canada | 11 | – | – | – | – | – | – | – | – | – | – |
| Egypt | 3 | – | – | – | – | – | – | – | – | – | – |
| Finland | 12 | 36 | 2 | 4 | 0 | 2.00 | 2 | 5 | 4 | 3.67 | +1.7 |
| France | 12 | – | – | – | – | – | – | – | – | – | – |
| Germany | 18 | – | – | – | – | – | – | – | – | – | – |
| India | 6 | 34 | 0 | 6 | 4 | 3.33 | 5 | 3 | 4 | 4.00 | +0.7 |
| Iran (Islamic Republic of) | 5 | 2 | 1 | 2 | 2 | 1.67 | 1 | 1 | 2 | 1.33 | –0.3 |
| Italy | 4 | 21 | 1 | 0 | 1 | 0.67 | 1 | 3 | 2 | 2.00 | +1.3 |
| Japan | 20 | 25 | 1 | 14 | 6 | 7.00 | 6 | 8 | 8 | 7.33 | +0.3 |
| Republic of Korea | 28 | – | – | – | – | – | – | – | – | – | – |
| Malaysia | 22 | 11 | 22 | 4 | 6 | 10.67 | 4 | 2 | 2 | 2.67 | –8.0 |
| Netherlands | 13 | 3 | 2 | 2 | 1 | 1.67 | 2 | 3 | 2 | 2.33 | +0.7 |
| Philippines | 10 | 39 | 0 | 2 | 3 | 1.67 | 3 | 5 | 4 | 4.00 | +2.3 |
| Portugal | 13 | – | – | – | – | – | – | – | – | – | – |
| Russian Federation | 7 | 39 | 1 | 2 | 3 | 2.00 | 1 | 4 | 3 | 2.67 | +0.7 |
| Singapore | 10 | 6 | 6 | 7 | 16 | 9.67 | 14 | 6 | 8 | 9.33 | –0.3 |
| Spain | 12 | 26 | 0 | 4 | 0 | 1.33 | 2 | 3 | 2 | 2.33 | +1.0 |
| Sri Lanka | 11 | – | – | – | – | – | – | – | – | – | – |
| Sweden | 12 | 28 | 4 | 1 | 1 | 2.00 | 2 | 3 | 2 | 2.33 | +0.3 |
| Switzerland | 18 | 4 | 2 | 2 | 2 | 2.00 | 0 | 3 | 3 | 2.0 | 0.0 |
| Thailand | 14 | 6 | 8 | 32 | 7 | 15.67 | 3 | 3 | 3 | 3.00 | –12.7 |
| Turkey | 6 | 5 | 1 | 1 | 0 | 0.67 | 2 | 1 | 1 | 1.33 | +0.7 |
| United Arab Emirates | 13 | 24 | 10 | 6 | 6 | 7.33 | 7 | 4 | 5 | 5.33 | –2.0 |
| The United Kingdom | 13 | – | – | – | – | – | – | – | – | – | – |
| United States of America | 15 | – | – | – | – | – | – | – | – | – | – |
| Viet Nam | 17 | 14 | 29 | 7 | 7 | 14.33 | 8 | – | – | 8.00 | –6.3 |
| **Median (IQR)** | **12 (10–17)** | – | – | – | – | – | – | – | – | – | – |
| **Mean** | – | **18** | – | – | – | **4.95** | – | – | – | – | – |

^a^Naming of countries and territories is in accordance with World Health Organization COVID-19 daily situation reports.

^b^Values in this column are actual SN values matching yellow cells in Table A2.

^c^Taking the median SN of 12, ST was observed in Table A2 as the number of days from the first case reported until the date closest to 12 cases without going over.

^d^Early epidemic DTs (ie, the first three DTs) were observed in Table A2 as the number of days starting from SN=12 cases to DT1 (24 cases), then to DT2 (48 cases), and then to DT3 (96 cases), getting as close as possible to these case counts each time without going over. Case counts are shown in red in Table A2.

^e^Later epidemic DTs (ie, the second three DTs) were observed in Table A2 as the number of days starting from DT3 (96 cases) to DT4 (192 cases), then to DT5 (384 cases), and then to DT6 (768 cases), getting as close as possible to these case counts each time without going over. Case counts are shown in blue in Table A2.

^f^Change in mean DT is calculated as later epidemic mean DT minus early epidemic mean DT.

**Table S4. ST and DT values used for all countries/territories assessed for risk using the ST/DT model.**

| **Country/Territory^a^** | **ST, days^b^** | **DT, days^c^** |
| --- | --- | --- |
| *European Region* |  |  |
| Albania | 5 | 3.67 |
| Andorra | 17 | 1.67 |
| Bulgaria | 7 | 2.00 |
| Bosnia and Herzegovina | 11 | 2.67 |
| Belarus | 14 | 6.33 |
| Cyprus | 6 | 3.00 |
| Faroe Islands | 9 | 2.00 |
| Georgia | 11 | 7.33 |
| Hungary | 7 | 3.33 |
| Kazakhstan | 5 | 3.00 |
| Kyrgyzstan | 3 | 3.33 |
| Latvia | 11 | 3.00 |
| Malta | 9 | 3.00 |
| Republic of Moldova | 9 | 3.00 |
| Montenegro | 4 | 3.33 |
| North Macedonia | 17 | 2.67 |
| San Marino | 7 | 3.67 |
| Slovakia | 8 | 1.67 |
| Uzbekistan | 4 | 3.33 |
| *Eastern Mediterranean Region* |  |  |
| Afghanistan | 17 | 4.33 |
| Jordan | 12 | 2.00 |
| Kuwait | 1 | 5.67 |
| Lebanon | 10 | 4.33 |
| Oman | 7 | 7.00 |
| occupied Palestinian territory | 3 | 7.00 |
| Tunisia | 12 | 3.33 |
| *Region of the Americas* |  |  |
| Bolivia (Plurinational State of) | 9 | 3.33 |
| Costa Rica | 6 | 3.33 |
| Cuba | 8 | 3.00 |
| Guadeloupe | 4 | 3.33 |
| Honduras | 10 | 3.00 |
| Martinique | 8 | 4.67 |
| Puerto Rico | 11 | 2.33 |
| Venezuela (Bolivarian Republic of) | 3 | 3.33 |
| Uruguay | 4 | 1.33 |
| *African Region* |  |  |
| Burkina Faso | 8 | 2.33 |
| Cameroon | 13 | 3.67 |
| Democratic Republic of the Congo | 10 | 4.00 |
| Côte d’Ivoire | 10 | 2.00 |
| Ghana | 8 | 2.00 |
| Mauritius | 2 | 2.33 |
| Nigeria | 22 | 3.00 |
| Réunion | 6 | 3.00 |
| Senegal | 12 | 4.00 |

^a^Naming of countries and territories is in accordance with World Health Organization COVID-19 daily situation reports.

^b^Taking the median SN of 12, ST values were the numbers of days from the first cases reported until the dates closest to 12 cases without going over.

^c^DT values were the numbers of days starting from SN=12 cases to DT1 (24 cases), then to DT2 (48 cases), and then to DT3 (96 cases), getting as close as possible to these case counts each time without going over.
